# Key changes in the future clinical application of ultra-high dose rate radiotherapy

**DOI:** 10.3389/fonc.2023.1244488

**Published:** 2023-10-24

**Authors:** Binwei Lin, Mi Fan, Tingting Niu, Yuwen Liang, Haonan Xu, Wenqiang Tang, Xiaobo Du

**Affiliations:** ^1^Department of Oncology, National Health Commission (NHC) Key Laboratory of Nuclear Technology Medical Transformation (Mianyang Central Hospital), Mianyang Central Hospital, School of Medicine, University of Electronic Science and Technology, Mianyang, China; ^2^Department of Oncology, Affiliated Hospital of North Sichuan Medical College, Nanchong, China

**Keywords:** FLASH-RT, clinical application, simulations, target delineation, treatment plan evaluation

## Abstract

Ultra-high dose rate radiotherapy (FLASH-RT) is an external beam radiotherapy strategy that uses an extremely high dose rate (≥40 Gy/s). Compared with conventional dose rate radiotherapy (≤0.1 Gy/s), the main advantage of FLASH-RT is that it can reduce damage of organs at risk surrounding the cancer and retain the anti-tumor effect. An important feature of FLASH-RT is that an extremely high dose rate leads to an extremely short treatment time; therefore, in clinical applications, the steps of radiotherapy may need to be adjusted. In this review, we discuss the selection of indications, simulations, target delineation, selection of radiotherapy technologies, and treatment plan evaluation for FLASH-RT to provide a theoretical basis for future research.

## 1 Introduction

Cancer is one of the main causes of death, and there were an estimated 19.3 million new cases of cancer and almost 10.0 million deaths from cancer worldwide in 2020 (1). Radiotherapy is an important local treatment strategy for cancer that effectively controls tumor growth and prolongs the patient’s survival time (2, 3). Moreover, radiotherapy can alleviate pain (4), obstruction (5), and bleeding (6) caused by cancer, thereby improving the quality of life of patients with cancer. However, the curative effects of radiotherapy remain limited (7). Insufficient doses to the tumor area may be an important cause of tumor recurrence after radiotherapy (7). Radiotherapy can cause damage to the organs at risk (OAR) around the cancer (8), and the dose of external radical radiotherapy is often limited to 60–70 Gy to avoid unacceptable toxicity (9, 10).

Ultra-high dose rate radiotherapy (FLASH-RT) is an external beam radiotherapy strategy that uses an extremely high dose rate (≥40 Gy/s) (11). Compared with conventional dose rate radiotherapy (COVN-RT) (≤0.1 Gy/s), the main advantage of FLASH-RT is that it can reduce damage to OARs surround the cancer (11) and retain the anti-tumor effect (12); this phenomenon is called the FLASH effect, which suggests that FLASH-RT may widen the treatment window (13). At present, different particle types (photon, electron and proton) are used in FLASH-RT (14). Many current FLASH-RT studies use electron linear accelerators (15, 16); however, due to the low tissue penetration and limited field size of electron beams, they cannot be used for the treatment of deep tumors. As proton beam and photon beam offer the greater tissue penetration depth and, therefore, allow irradiation of deep-seated tumors, they are both considered as the most promising for clinical application (11, 14, 17). Furthermore, due to the presence of the Bragg peak, the proton beam may have a better dose distribution than photon beam, but the cost of a proton beam is more expensive (18). Oxygen depletion is one of the hypotheses for the mechanism of FLASH effect. As a single fraction of FLASH-RT may complete the irradiation in an extremely short time, the oxygen in the tissue is rapidly exhausted, and it is too fast to be supplemented by the circulating blood. This results in a relative hypoxia in the tissue compared with that following COVN-RT (irradiation completed in a few minutes), which may be one of the reasons why FLASH-RT can protect the normal tissue (19). However, the mechanism of the FLASH effect is unclear, and we discussed it in detail in our previous work (20).

Preclinical studies have demonstrated the protective advantages of FLASH-RT in normal tissues of the lungs (12), intestine (21), and brain (22). The first clinical study on FLASH-RT was reported in 2019 (23). In this study, a patient with skin T-cell lymphoma received FLASH-RT (electron, 166.7 Gy/s, a single total dose of 15 Gy); the tumor in the radiation area reached complete remission, and only grade 1 skin toxicity occurred (23). Recently, the results of the FAST-01 clinical study were published; ten patients with bone metastases in the extremities received a total dose of 8 Gy of FLASH-RT (51–61 Gy/s). The pain relief rate was 67% (complete relief rate, 50%), and no grade 3 treatment-related toxicity was observed (24). Owing to the safety and effectiveness of FLASH-RT for the treatment of bone metastasis in the FAST-01 study, the FAST-02 study began to recruit patients in 2022. However, in the FAST-01 study, only metastases in extremities were included, and a single rectangular field (from 7.5 cm × 7.5 cm up to 7.5 cm × 20 cm) was used to treat bone metastases. When the indications were expanded to body tumors with a more complex anatomical structure, it was difficult to meet the clinical requirements. In addition, due to the extremely short irradiation time and limitations on single total dose and dose rate of FLASH-RT, the traditional radiotherapy treatment process may not be applicable to FLASH-RT. Therefore, problems in the clinical implementation of FLASH-RT need to be further explored. In this review, we discuss the problems that need to be considered in the process of FLASH-RT clinical implementation, such as indication selection, stimulation, target delineation, selection of radiotherapy technology, and treatment plan evaluation, to provide a theoretical basis for future research. It should be noted that quality assurance is another important issue in clinical applications of FLASH-RT, which is not discussed in this review.

## 2 Indication

As reported in current preclinical studies, to trigger the FLASH effect, it may be necessary to simultaneously achieve both ultra-high dose rate (≥40 Gy/s) and large single dose per fraction (19, 25). An *in vitro* study showed that when a single dose reached 20 Gy, FLASH-RT reduced DNA damage in lung fibroblasts and increased the cell survival rate. When a single dose was <20 Gy, the protective effect of FLASH-RT disappeared (26). Although the single-dose threshold of FLASH-RT to achieve the FLASH effect may be different in different normal tissues (**Table 1**), we can speculate that in clinical applications, FLASH-RT may only be suitable for high-dose fractionated radiotherapy. Therefore, the experience gained from the current clinical use of stereotactic radiotherapy (SBRT) technology may provide useful experience for the clinical implementation of FLASH-RT, and tumors suitable for SBRT treatment, such as non-metastatic lung cancer (57), liver cancer (58), brain cancer (59), and oligometastases in the lung, liver, may be indications for FLASH-RT (60, 61). However, we need to consider the tumor location and treatment purpose during clinical experiments with FLASH-RT.

**Table 1:** Summary of the dose and dose rate used in published studies of FLASH-RT.

System	Vitro/vivo	Author(s)	Year	Modal	Radiation source	Total dose/fractions	Dose rate (Gy/s)	Protective effect*
Lung	*In vivo*	Favaudon V (12)	2014	mice	electron	17~30Gy/1F	60	Yes
		Fouillade C (27)	2020	mice	electron	17Gy/1F	60	Yes
		Feng G (28)	2022	mice	photon	30Gy/1F	700~1200	Yes
	*In vitro*	Buonanno M (26)	2019	lung fibroblasts	proton	20Gy/1F	100 or 1000	Yes
						5, 10 or 15Gy/1F	100 or 1000	No
		Adrian G (29)	2021	lung fibroblasts	electron	2~10Gy/1F	≥800	No
		Fouillade C (27)	2020	lung fibroblasts	electron	4Gy/1F	60	Yes
						2Gy/1F	60	No
		Guo Z (30)	2022	lung fibroblasts	proton	unkown	100	Yes
Brain	*In vivo*	Montay-Gruel P (31)	2017	mice	electron	10Gy/1F	≥30	Yes
						10Gy/1F	≤20	No
		Montay-Gruel P (32)	2018	mice	electron	10Gy/1F	37	Yes
		Simmons DA (33)	2019	mice	electron	30Gy/1F	200 or 300	Yes
		Montay-Gruel P (34)	2019	mice	electron	10Gy/1F	>100	Yes
		Montay-Gruel P (22)	2020	mice	electron	10Gy/1F	5.6×10^6^	Yes
		Alaghband Y (35)	2020	mice	electron	8Gy/1F	4.4×10^6^	Yes
		Allen BD (36)	2020	mice	electron	10 or 25 Gy/1F	2,500 or 5.6×10^6^	Yes
		Montay-Gruel P (37)	2021	mice	electron	10Gy/1F	5.6×10^6^	Yes
						14Gy/1F	7.8×10^6^	No
						14Gy/2F	3.9×10^6^	Yes
						14Gy/4F	1.9×10^6^	No
						30Gy/3F	5.6×10^6^	Yes
						25Gy/1F	2.5×10^3^	No
		Dokic I (38)	2022	mice	proton	10Gy/1F	120	Yes
Intestine	*In vivo*	Levy K (39)	2020	mice	electron	14 or 16Gy/1F	216	Yes
		Ruan JL (21)	2021	mice	electron	7.5~12.5Gy/1F	2.2~5.9×10^6^	Yes
						11.2 or 12.5Gy/1F	≥280	Yes
						11.2 or 12.5Gy/1F	<280	No
		Kim MM (40)	2021	mice	proton	15 or18Gy/1F	106.2~118.5	Yes
		Feng G (28)	2022	mice	photon	12Gy/1F	700~1200	Yes
		Shi X (41)	2022	mice	photon	13Gy/1F	110~120	Yes
		Zhu H (42)	2022	mice	photon	10 or 15Gy/1F	>150	Yes
		Zhang Q (43)	2023	mice	proton	14~18Gy/1F	120	No
Skin	*In vivo*	Vozenin MC (44)	2019	mini pig/cat	electron	22~41Gy/1F	≈300	Yes
		Soto LA (45)	2020	mice	electron	30 or 40Gy/1F	180	Yes
						10, 16 or 20Gy/1F	180	No
		Singers Sørensen B (46)	2021	mice	proton	31.2~53.5Gy/1F	65~92	Yes
		Velalopoulou A (47)	2021	mice	proton	30 or 45Gy/1F	69-124	Yes
		Gaide O (48)	2022	human patient	electron	15Gy/1F	167	No
		Miles D (49)	2023	Mice	X-rays	35Gy/1F	87	Yes
						43Gy/1F	87	No
immune system	*In vitro*	Bozhenko VK (50)	2019	normal lymphocytes	photon	1~4Gy/1F	1.7~6.7×10^7^	Yes
	–	Jin JY (51)	2020	computation study	–	2Gy/1F	0.0017~333	No
						>2~50 Gy/1F	<40	No
						>2~50 Gy/1F	≥40	Yes
Others	*In vivo*	Beyreuther E (52)	2019	zebrafish	proton	>15~40Gy/1F	100	Yes
						≤15 Gy/1F	100	No
		Ohsawa D (53)	2022	plasmid DNA	proton	19.6~97Gy/1F	40	Yes
		Eggold JT (54)	2022	mice	electron	14Gy	210	Yes
		Karsch L (55)	2023	zebrafish	electron/proton	30.1~32.3Gy/1F	177.2~2.5×10^5^	Yes
		Cuitiño MC (56)	2023	mice	electron	5~16Gy/1F	234; 2.35×10^6^; 2.31×10^6^	No

*Compare with conventional dose rate radiotherapy.

First, noncavitary organs may be more suitable for FLASH-RT than cavitary organs. This is because when high-dose fractionated radiotherapy is used for cavitary organ tumors, rapid shrinkage of the tumor may lead to organ perforation (e.g., esophageal perforation), infection, bleeding, or even death (62). The tumor size may also affect the implementation of FLASH-RT because if the tumor size is too large, OARs often have difficulty tolerating a single high-dose irradiation (63). However, further research is needed to determine the limitation of the tumor size on FLASH-RT.

Second, the purpose of treatment (palliative radiotherapy, radical radiotherapy, neoadjuvant radiotherapy, or adjuvant radiotherapy) is also an important factor in the selection of indications. The purpose of palliative radiotherapy is to relieve pain, bleeding, obstruction, and other symptoms at a low total dose, rather than to completely kill the tumor. Palliative radiotherapy is often used in the late stage of cancer, and its main goal is to improve the patient’s quality of life. A low total dose can maximize patient safety; therefore, in the early stage of the clinical implementation of FLASH-RT, palliative treatment should first be selected as an indication, as in the FAST-01 study (24). Follow-up clinical research is needed to explore the efficacy and safety of FLASH-RT in the treatment of pain, tumor hemorrhage, and tumor obstruction caused by tumor metastasis to the trunk. In 2022, FAST-02 was launched, the main inclusion criterion was patients with chest bone metastasis, which will provide important insights for the clinical application of FLASH-RT in palliative treatment. Radical radiotherapy refers to the use of a higher total dose as the main local treatment method to completely kill the tumor, and the main purpose of radical radiotherapy is to cure cancer and prolong the survival time. Because FLASH-RT may widen the treatment window of radiotherapy and a higher target dose could be delivered to the tumor, it is of great relevance to evaluate whether FLASH-RT can improve the anti-tumor effect; therefore, it is of great importance to evaluate the efficacy and safety of FLASH-RT in radical radiotherapy. Neoadjuvant and adjuvant radiotherapies are often used as auxiliary means of surgical treatment before (neoadjuvant radiotherapy) or after (adjuvant radiotherapy) surgery. The main purpose of neoadjuvant or adjuvant radiotherapy is to improve the antitumor effect of surgery and reduce the risk of recurrence. However, in neoadjuvant or adjuvant radiotherapy, irradiation of the lymph node drainage area is typically considered when dealing with regional microscopic tumor spread or incomplete resection (64, 65), which means that the treatment target area is too large to use single high-dose irradiation. However, there were no reports of using FLASH-RT for treatment of lymphatic node drainage areas. In order to determine whether the lymph node drainage area is suitable for FLASH-RT, future research needs to clarify two points: 1) the maximum tolerable dose of OARs around the lymph node drainage area under ultra-high dose rate irradiation condition; 2) the maximum tolerable dose that can be tolerated by OARs large enough to trigger the FLASH effect.

## 3 Radiotherapy simulation

Radiotherapy simulation is an important preparation step before radiotherapy. Its main purpose is to obtain a repeatable matching three-dimensional computed tomography (CT) image of the patient to meet the target area delineation and implement accurate radiotherapy (66). Bourhis et al. reported the first human FLASH-RT study in 2019. In this study, ultra-high dose rate electron was used treat a patient with cutaneous T-cell lymphoma, and the treatment area is limited by the collimator; therefore, no simulation was conducted (23). FAST-01 study was the first study that reported the experience of FLASH-RT simulation (24). After fitting with an immobilization device, each participant accepted CT simulation imaging for the area(s) encompassing the treatment targets (24). However, FAST-01 study only included patients with bone metastases in the extremities, the target area was fixed, and this experience of FLASH-RT simulation cannot be applied to situations where the target area is movable. Similar to SBRT, the radiotherapy simulation of FLASH-RT requires postural fixation. Thermoplastics is a mature tool that ensures the relative consistency of a patient’s position during positioning and treatment (67). However, movement of internal organs during treatment, such as pharyngeal swallowing activity, respiratory movement, gastrointestinal motility and heartbeat, is an important factor affecting the accuracy of radiotherapy (68–70). Because of the long implementation time of SBRT (minutes to tens of minutes) (71, 72), it is unlikely the movement of organs in the body near the target area can be avoided. Consequently, it is often necessary to expand the volume of the target area to cover the movement track of the tumor target area and avoid the tumor omission (73). CyberKnife is a special treatment platform for SBRT, and its organ tracking function is an important advantage (74). Organ movement can be simulated to the maximum extent using reference marks implanted on the body surface or *in vivo*. Subsequently, the position is matched using a 6D treatment couch to reduce the volume expansion of the target area caused by organ movement (74). However, the treatment time of FLASH-RT is extremely short (within milliseconds) (11). The organ position may remain unchanged during FLASH-RT irradiation, so FLASH-RT may not need to simulate the dynamic process of organ movement. The problem that needs to be addressed is whether the tumor location is consistent with the initial CT location. Therefore, it may be necessary to implant a target reference object (e.g., a metal marker) into the tumor before simulation. Moreover, an auxiliary device similar to an “adaptive switch” is needed to trigger FLASH-RT immediately when the tumor moves to the target position. By ignoring the effects of organ movement on the target location, FLASH-RT may further narrow the target area to improve normal tissue sparing. The feasibility of implanting markers into tumors and using position matching to trigger FLASH-RT should be verified in subsequent studies. However, marks implantation is an invasive procedure, and although the accuracy of treatment is guaranteed, it may bring risks of pain, bleeding, infection, and tumor metastasis. Therefore, a safer radiotherapy simulation mode may need further exploration.

## 4 Target delineation

Target delineation is a step for clinicians to determine the scope of the tumor and to delineate the OARs around the target area through clinical physical examination, imaging data, endoscopic data, and tumor biological behavior. This process determines the radiotherapy treatment area (tumor) and protection area (OARs) (75). In traditional radiotherapy, the target areas to be delineated include the gross tumor volume, clinical target volume (CTV), internal target volume (ITV), and planning target volume (76). Considering the extremely short delivery time and dose-rate threshold of FLASH-RT, we propose the following target delineation approaches.

### 4.1 ITV delineation may not be necessary

The ITV refers to the expanded margin when the position of the CTV is uncertain (mainly due to organ movement, filling, and deformation) (76). Due to the FLASH-RT treatment time being extremely short and internal organ movement in the treatment process having little impact on the target location, once the tumor location is consistent with the initial CT location, FLASH-RT will be triggered. As a result, the ITV may not need to be delineated. Deletion of the ITV may be particularly suitable for FLASH-RT in liver and lung tumors affected by respiratory movement. In future research, more evidence and clinical data are needed to support the feasibility of omitting the ITV. However, when the target area is close to the heart or large blood vessels, and tachycardia is existing simultaneously, the ITV may not be omitted. For example, when using a total dose of 20 Gy and a dose rate of 40 Gy/s to treat a patient with a heart rate of 120 beats/min, the time for a single irradiation is half a second, and the patient’s heart can beat once in half a second, indicating that the heartbeat still affects the position of the tumor target area. A 4D cardiac dual-source CT may be a useful tool to obtain the moving boundaries of the heart and guide the delineation of ITV in this situation (77).

### 4.2 Addition of the dose rate organs at risk may be needed

**Figure 1:**
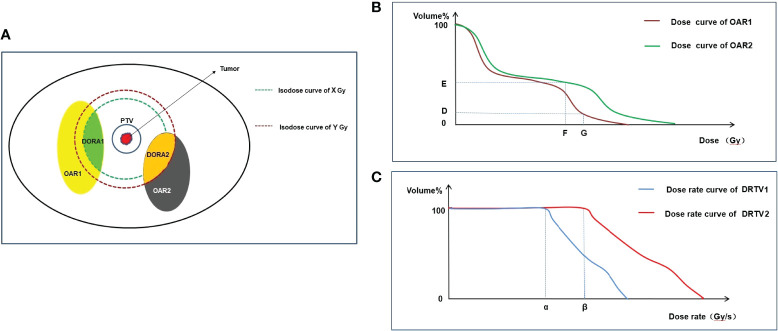
Graphical representation of DOARs delineation and TPE. OAR1 and OAR2 represent two normal organs around tumor **(A)**, respectively. Firstly, dose optimization is performed and evaluated using DVH to meet the following requirements: in OAR1, V_G_<D%, and in OAR2, V_F_<E% **(B)**. Secondly, after dose optimization is completed, DOARs are delineated **(A)**. X Gy and Y Gy are the threshold doses that trigger the FLASH effect of OAR1 and OAR2, respectively. In OAR1, the area (green) with dose ≥A Gy may produce the FLASH effect, and the dose rate assessment is required, which is defined as DOAR1. In OAR2, the area (orange) with dose ≥B Gy may produce the FLASH effect, and the dose rate assessment is required, which is defined as DOAR2. Dose rate is evaluated by DRVH **(C)**. α and β are the threshold dose rates that trigger the FLASH effect of OAR1 and OAR2, respectively. To trigger the FLASH effect, the dose rate of αshould cover 100% volume of DOAR1 and the dose rate of β should cover 100% volume of DOAR2. DOARs, dose rate organs at risk; OARs, organs at risk; DVH, dose–volume histogram; DRVH, dose rate–volume histogram; TPE, treatment plan evaluation.

As the protective effect of FLASH-RT requires the dose rate of radiotherapy to reach 40 Gy/s or higher, OARs must meet this dose-rate threshold. However, tumors must be killed rather than protected, and the dose rate of the tumor areas does not have to reach the threshold to trigger the FLASH effect. To evaluate the scope of the area to be protected by FLASH-RT more accurately, the dose rate organs at risk (DOARs) may need to be defined to facilitate treatment planning. Considering that a threshold dose is needed to trigger the FLASH effect and that the lowest single dose to trigger the FLASH effect in different OARs may be inconsistent (**Table 1**), areas in OARs that absorbed doses lower than the threshold dose to trigger the FLASH effect may not be required to reach 40 Gy/s or above. Therefore, it may not be possible to define OARs directly as the DOARs. The DOARs is defined as the area in the OARs that exceeds the minimum dose threshold (i.e., the minimum dose that triggers the FLASH effect) (**Figure 1A**).

The dose distribution of OARs can only be obtained after the completion of radiotherapy dose optimization. Therefore, the delineation of the DOARs should be sketched after completion of the first treatment plan (dose optimization), and the second step of the treatment plan (dose rate optimization) should be subsequently conducted. However, reaching the dose-rate threshold of FLASH-RT requires extremely high-technology radiotherapy equipment, which results in high costs (78). Adding the DOARs to OARs in the treatment field and selectively meeting the dose-rate threshold of FLASH-RT may further reduce the technical difficulty of the clinical implementation of FLASH-RT. Moreover, the DOARs will facilitate clinicians in evaluating treatment plans in more dimensions; this is discussed in detail in the plan evaluation section.

## 5 Radiotherapy technology

In traditional radiotherapy, radiotherapy technologies include two-dimensional radiotherapy (2DRT), three-dimensional conformal radiotherapy (3DCRT), intensity-modulated radiotherapy (IMRT), and image-guided radiotherapy (IGRT) (79). In the case of a simple target structure and a few surrounding OARs (e.g., limb bone metastasis), 2DRT or 3DCRT can meet the treatment requirements. However, when the shape of the target area is complex, particularly with a groove structure, 2DRT or 3DCRT often cannot produce a highly conformal dose distribution area (76, 80). Therefore, IMRT is currently the most widely used radiotherapy technique in clinical practice (81). IMRT can form multiple subfields in any direction by moving a multileaf collimator (MLC) to optimize the dose distribution in the target area (82). However, in the process of producing a daughter field, the MLC movement requires sufficient time (83), which prolongs the treatment time and reduces the average dose rate of radiotherapy, resulting in failure to meet the dose-rate threshold of FLASH-RT. Therefore, IMRT is not applicable to FLASH-RT, but 3D-CRT may be a radiotherapy technology suitable for FLASH-RT. At the same time, in the process of FLASH-RT, it is necessary to accurately locate the tumor before treatment; hence, IGRT is necessary, but it is impossible to use IGRT for real-time position adjustment in the treatment because the FLASH-RT treatment time is very short and the time required for image matching and treatment bed movement far exceeds the time of FLASH-RT.

## 6 Treatment plan evaluation

Treatment plan evaluation (TPE) is an important step before the implementation of radiotherapy. The main purpose of TPE is to evaluate whether the target areas in the radiotherapy plan reach the prescribed dose, the uniformity and conformability of the target area, the dose hot and cold spots in the target area, and whether the dose of OARs exceeds the limit value (76). In traditional radiotherapy, the isodose curve and dose–volume histogram (DVH) are important evaluation tools (84, 85). However, in FLASH-RT, the dose rate is an important physical parameter because a normal tissue protection effect can be achieved only when the dose rate exceeds a threshold. Therefore, we propose that additional tools should be provided when evaluating normal tissues, including the isodose rate curve and dose rate–volume histogram (DRVH).

### 6.1 Isodose rate curve

The isodose rate curve refers to the curve connected by the voxels that receive the same dose rate in a three-dimensional human-simulated CT image. In traditional radiotherapy, the isodose curve can assist clinicians in evaluating the coverage of OARs at a certain dose in the axial, sagittal, and coronal positions. Take the spinal cord as an example, in order to meet the limit that the maximum dose cannot exceed 50 Gy (conventional fraction) (86), an isodose rate curve of 50 Gy should not include the spinal cord. Similarly, when evaluating the DOARs, the lowest dose rate isodose rate curve that triggers the FLASH effect must include all the DOARs. When evaluating OARs, the dose-rate curve is an important supplement to the dose curve. The dose curve can evaluate the radiation dose of normal tissues to meet the dose limit of traditional radiotherapy on normal tissues; the dose-rate curve can give full play to the technical advantages of FLASH-RT and make use of the protective effect of the FLASH dose rate on normal tissues to protect normal tissues more effectively and reduce the incidence of toxicity. However, because the minimum dose-rate threshold for triggering the FLASH effect in different tissues may be inconsistent (**Table 1**), the isodose rate curves of interest may also be inconsistent when evaluating different OARs. In future studies, a large number of preclinical and clinical trials are required to determine the minimum dose rate for different OARs.

### 6.2 DRVH

DRVH is a statistical chart that evaluates the proportion of the fixed dose rate volume in the overall volume in DRVH with a dose rate as the horizontal axis and volume percentage as the vertical axis (**Figures 1B, C**). In traditional radiotherapy, the isodose curve is often only able to determine whether the region of interest is covered by a certain dose curve, but the extent of coverage cannot be provided by the isodose curve; therefore, the DVH tool is needed for further evaluation. DVH is an important evaluation tool for parallel organs (e.g., the lungs and kidneys) (86). For example, V20 (the proportion of the lung volume covered by a dose of >20 Gy to the total lung volume) is significantly associated with the incidence of radiation pneumonia. In TPE of the lung, V20 <30% is used in clinical practice to limit the incidence of radiation-induced lung injury (87). Similarly, the isodose rate curve alone cannot indicate the extent to which the region of interest is covered by the target dose rate curve. Therefore, it may be necessary to introduce DRVH to obtain additional information. More importantly, the dose-rate curve histogram can quantify the coverage of the dose-rate curve of OARs, which is helpful in analyzing the relationship between the dose-rate volume and the incidence of adverse reactions and can guide follow-up clinical practice. **Figure 1** is used to illustrate the practical use of DVH and DRVH in TPE. It should be noted that we have only speculated theoretically about the TPE tools of FLASH-RT. The prerequisite for the application of these tools is to clarify the physical conditions that trigger the FLASH effect (dose rate, total single dose), as well as the maximum tolerable dose of OARs under ultra-high dose rate conditions. However, these physical parameters are currently unclear and need to be clarified in future biological experiments.

## 7 Implement of FLASH-RT

Currently, only two clinical studies have reported the implementation of FLASH-RT (23, 24), however, both of these clinical studies used surface markers for setup and treatment. For surface or limb lesions with fixed tumor locations, this method is acceptable, but for deep tumors, especially those with organ movements, it is necessary to explore a real-time imaging method to ensure that dose is accurately transmitted to the tumor area. Currently, the mature technology used in the implementation of COVN-RT is CBCT image-guided radiotherapy (88). Patients usually need to undergo CBCT scanning after fixation, and the obtained images are matched with CT-simulated images for position registration. An error range of less than 3 mm is often considered an acceptable range (89). However, the slow acquisition time of CBCT images would limit its utility for real-time imaging. Recently, El Naqa et al. proposed that MRI or ultrasound images may be suitable for real-time image guidance of FLASH-RT (90). MRI-guided radiotherapy has become more popular in recent years as it has superior soft tissue contrast and no moving parts for 3D imaging, allowing for real-time motion monitoring (91). Ultrasound provides a more economical, non-invasive, real-time, and radiation free imaging method that can provide (even time-resolved) 3D-US anatomical visualization for selected anatomical positions. Meanwhile, ultrasound has been used in the past for image-guided radiotherapy of prostate cancer and gynecological cancer (92). In future research, it is necessary to further verify the feasibility of applying these two image-guided technologies to FLASH-RT.

## 8 Conclusions

**Figure 2:**
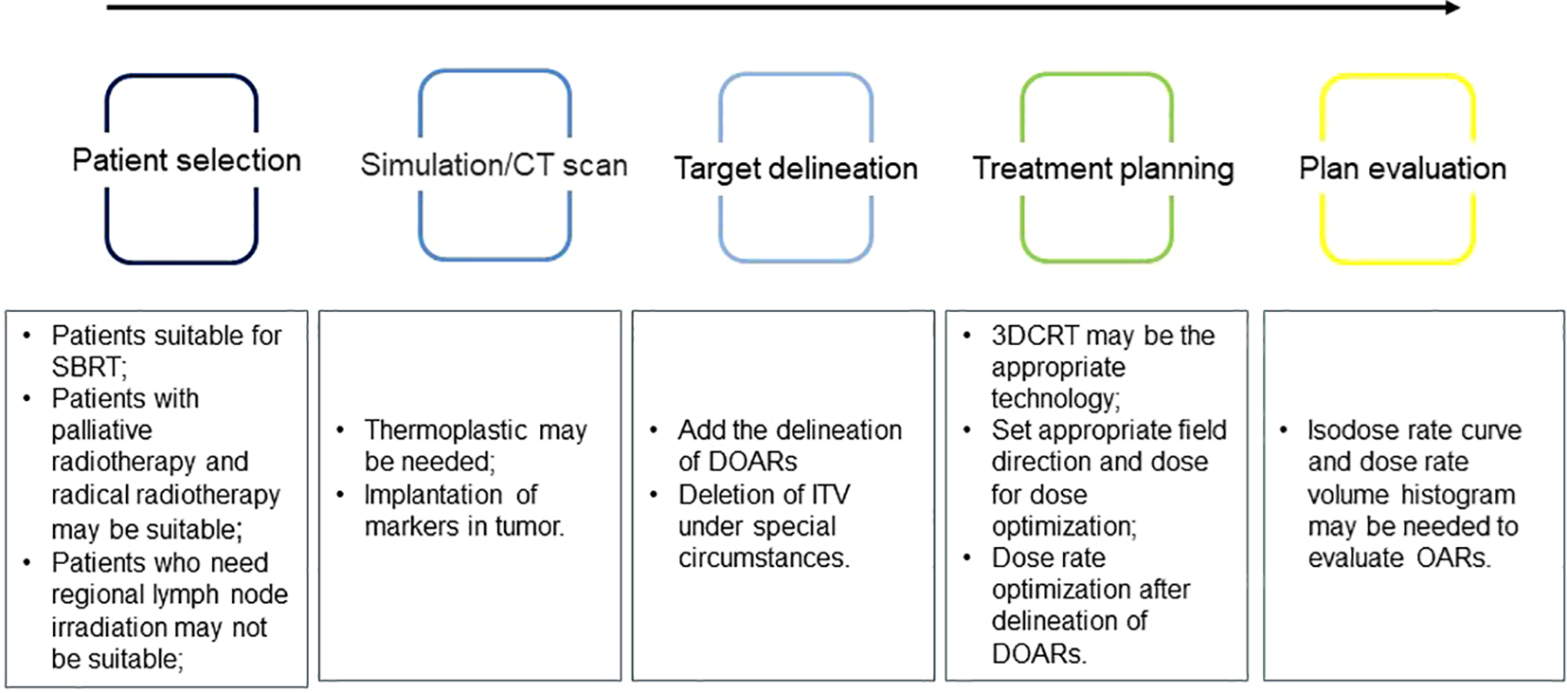
Problems that may need attention during the implementation of ultra-high dose rate radiotherapy.

This review discusses the changes in the future clinical application of FLASH-RT and provides new research directions for the clinical transformation of FLASH-RT. First, we discussed the indications for FLASH-RT and suggested that tumors suitable for SBRT may be suitable for FLASH-RT. Simultaneously, the radiotherapy process may need to be modified (**Figure 2**). The ITV may not be required for target area delineation, and for normal tissues, DOARs may need to be added. Because of the long modulation time of IMRT, 3D-CRT may be a suitable radiotherapy technology for FLASH-RT. When conducting TPE, it may be necessary to introduce a dose-rate curve and DRVH to fully evaluate whether OARs have received sufficient dose-rate radiation to achieve a better protective effect. However, the clinical application of FLASH-RT requires further clinical trials to ensure better validation and improvement, especially in the treatment of irregularly shaped tumors in the body. It may be challenging to ensure that the radiation dose is accurately transmitted to the tumor target area and achieve sufficient dose rate and total single dose to trigger the FLASH effect.

## Data availability statement

The original contributions presented in the study are included in the article/supplementary material. Further inquiries can be directed to the corresponding author.

## Author contributions

BL and MF draft the manuscript, TN, YL, HX and WT participated in the data review and collection for the study. XD conceived of the study, revised and prepared the manuscript. All authors contributed to the article and approved the submitted version.
